# The association of childhood maltreatment with depression and anxiety is not moderated by the oxytocin receptor gene

**DOI:** 10.1007/s00406-017-0784-z

**Published:** 2017-03-28

**Authors:** Marieke S. Tollenaar, Marc L. Molendijk, Brenda W. J. H. Penninx, Yuri Milaneschi, Niki Antypa

**Affiliations:** 1 Department of Clinical Psychology, Institute of Psychology, Leiden Institute for Brain and Cognition, Leiden University, P.O. Box 9555, 2300 RB Leiden, The Netherlands; 2 Department of Psychiatry, EMGO Institute for Health and Care Research and Neuroscience Campus Amsterdam, VU University Medical Center/GGZ inGeest, Amsterdam, The Netherlands

**Keywords:** OXTR, Oxytocin, Single nucleotide polymorphism, Anxiety, Depression, Childhood maltreatment

## Abstract

**Background:**

The oxytocin receptor (OXTR) gene may be involved in resilience or vulnerability towards stress, and hence in the development of stress-related disorders. There are indications that OXTR single nucleotide polymorphisms (SNPs) interact with early life stressors in predicting levels of depression and anxiety. To replicate and extend these findings, we examined whether three literature-based OXTR SNPs (rs2254298, rs53576, rs2268498) interact with childhood maltreatment in the development of clinically diagnosed depression and anxiety disorders.

**Methods:**

We included 2567 individuals from the Netherlands Study of Depression and Anxiety. This sample consisted of 387 healthy controls, 428 people with a current or past depressive disorder, 243 people with a current or past anxiety disorder, and 1509 people with both lifetime depression and anxiety diagnoses. Childhood maltreatment was measured with both an interview and via self-report. Additional questionnaires measured depression and anxiety sensitivity.

**Results:**

Childhood maltreatment was strongly associated with both lifetime depression and anxiety diagnoses, as well as with depression and anxiety sensitivity. However, the OXTR SNPs did not moderate these associations nor had main effects on outcomes.

**Conclusions:**

The three OXTR gene SNPs did not interact with childhood maltreatment in predicting lifetime depression and anxiety diagnoses or sensitivity. This stresses the importance of replication studies with regard to OXTR gene variants in general populations as well as in clearly established clinical samples.

## Introduction

Childhood maltreatment, including physical, sexual and emotional abuse, as well as physical and emotional neglect, has repeatedly been shown to be a strong risk factor for the development of stress-related disorders like depression and anxiety [1–3]. However, not all individuals that have experienced childhood maltreatment develop mental disorders in later life, indicating the presence of resilience or vulnerability factors that influence the consequences of such negative life experiences. Genetic and (neuro)biological factors that are involved in the processing of stress may play a role in the resilience and vulnerability to major life stressors [4]. In the current study we focus on the oxytocin system.

Oxytocin is a neuropeptide long known to be involved in birth and lactation, and in recent decades its role in human social behavior has been studied extensively. It has been associated with emotional bonding between parents and infants, trust, generosity and empathy, and is found to reduce anxiety in stressful situations [5–7]. Individual differences exist in basal oxytocin levels, and in oxytocin and oxytocin receptor (OXTR) gene polymorphisms. Human brain-imaging studies have shown that variations in OXTR gene polymorphisms are related to structural and functional variations in the amygdala, hypothalamus and cingulate gyrus, which are all involved in the processing of emotions and stress [8]. OXTR gene variations have often been linked to autism spectrum disorders [9], but the OXTR gene may be involved in the development and/ or maintenance of anxiety and depressive disorders as well.

Genome wide association studies (GWAS), including a mega-analysis, have not yielded any direct associations between OXTR gene polymorphisms and depressive disorders [10, 11]. One small candidate gene study did report evidence for involvement of OXTR gene single nucleotide polymorphisms (SNPs) rs2254298 and rs53576 in clinically diagnosed major depressive disorder [12]. With regard to anxiety disorders (including generalized anxiety disorder, panic disorder and phobias), only a few GWAS and candidate gene studies have been reported in clinical samples so far [13, 14], with no report of any direct associations with the OXTR gene. Most evidence so far for involvement of the OXTR gene in both anxiety and depression comes from candidate gene studies looking at associations between OXTR SNPs and (endo-)phenotypes of anxiety and depression. That is, associations between the OXTR gene and personality or cognitive emotional characteristics that convey vulnerability for stress-related disorders have been found (e.g. [15–21]). These findings also suggest a possible common genetic involvement of the OXTR gene in the vulnerability to develop depression and anxiety.

Furthermore, there have been studies examining associations between OXTR gene polymorphisms and levels of anxiety and depression in community samples [22–25]. Interestingly, in all of these studies the OXTR gene polymorphisms interacted with stressful life experiences in predicting levels of anxiety or depression. That is, Thompson et al. [25] showed that OXTR SNP rs2254298 interacted with adverse parental environment to predict symptoms of depression and anxiety in a sample of 92 Caucasian adolescent girls. More recent findings show that another OXTR SNP (rs53576) moderated the association between maternal depression in early childhood and depressive symptoms in a sample of 441 adolescents [23]. Similarly, McQuaid et al. [24] showed that in a sample of 288 students, early-life maltreatment was associated with depressive symptoms, and that the OXTR SNP rs53576 moderated this relationship. Furthermore, Myers et al. [22] found early life trauma to interact with OXTR SNPs in predicting levels of anxiety, stress and depression in a sample of 653 individuals. Hence, the effects of early life stress on depression and anxiety may depend on variants of the OXTR gene, indicating that certain OXTR SNPs confer differential susceptibility to stressful early life environments.

These prior studies examining OXTR polymorphisms in interaction with early life stress have used relatively small sample sizes. Replication of these findings hence merits consideration, also bearing in mind that replications of gene–environment interaction effects in other genetic domains, like the serotonergic system, have been inconsistent [26–28]. Furthermore, none of these studies examined clinically diagnosed depression and anxiety disorders. Therefore, our intention is to investigate in a large Caucasian sample from the Netherlands Study of Depression and Anxiety (NESDA), whether OXTR SNPs interact with experiences of childhood maltreatment in predicting clinically diagnosed lifetime depression and/ or anxiety disorders. Based on previous findings in this area, as discussed above [12, 22–25], the OXTR SNPs rs2254298 and rs53576 will be examined. In addition, the OXTR SNPs rs1042778 and rs2268498, which are thought to be involved in social behavior and negative emotionality [8, 21, 44], will be included as well. To increase the reliability of the measurement of childhood maltreatment, both interview-based and self-report measures are included. Also, in addition to the clinical diagnoses, measures of depression and anxiety sensitivity are used as outcomes measures as well to more closely replicate previous findings on vulnerability factors for anxiety and depression.

## Methods and materials

### Sample selection

Participants for our analyses were derived from the Netherlands Study of Depression and Anxiety (NESDA). NESDA is an ongoing prospective cohort study (*N* = 2981). For this study subjects with current depressive and/or anxiety disorders, patients with remitted depressive and/or anxiety disorders, and lifetime healthy controls were recruited from specialized mental health care, primary care, and the general population. An inclusion criterion was an age of 18 through 65 years. Excluded were individuals with a primary diagnosis of psychotic, obsessive compulsive, bipolar, or severe addiction disorder, and not being fluent in Dutch. For further details on the design, objectives, and protocol of NESDA we refer to Penninx et al. [29]. The study protocol was approved by the Ethical Review Board of the VU University Medical Center and by local review boards of each participating institute. After full information about the study was provided, written informed consent was obtained from all participants.

Our selection from the NESDA sample was based on the following criteria: (1) participants had to have a current or preceding (i.e. lifetime) diagnosis of depressive and/or anxiety disorders (*n* = 2180), or had never been diagnosed with either disorder (*n* = 387), including data from all prospective NESDA waves, covering 6 years of follow-up; (2) genomic data had to be available; and (3) participants had to be of North-European descent, which was based on reported birth country and nationality, and confirmed with ancestry-informative principal components (PC).

### Measures

Childhood Maltreatment (CM) was assessed retrospectively during the first NESDA wave, using a semi-structured childhood trauma interview, previously used in the Netherlands Mental Health Survey and Incidence Study [1]. In this interview, participants are asked whether they have experienced emotional neglect, emotional, physical and/or sexual abuse before the age of 16 years. Emotional neglect was described as “Nobody ever listened to you at home, your problems were ignored, or you had the feeling not being able to find any attention or support from your parents.” Emotional abuse was described as “You were verbally abused, unjustly punished, or your brothers and sisters were favored, but no bodily harm was done.” Physical abuse was defined as “Being kicked, hit with or without an object, or being physically maltreated in any other way.” Sexual abuse was defined as “Being touched sexually by anyone against your will, or being forced to touch anyone sexually.” After an affirmative answer, details on the frequency of these events and the perpetrators involved were asked for. Full data on this semi-structured childhood trauma interview was missing for 9 persons. For any type of CM, if it happened more than once, it was coded as present. The mean number (±SD) of CM types was 0.90 (±1.16), with 53.6% (*n* = 1376) reporting no CM, 18.8% (*n* = 481) reporting one type of CM, 14.9% (*n* = 382) reporting two types of CM, 9.0% (*n* = 231) reporting three types of CM, and 3.4% (*n* = 88) reporting 4 types of CM. For the main statistical analyses, the presence of CM was defined as 0 versus ≥1 type of CM.

In addition to the childhood trauma interview, participants also filled in a retrospective self-report questionnaire on the experience of CM, the Childhood Trauma Questionnaire (CTQ) short-form [30, 31] during the fourth NESDA wave, 4 years after baseline. The CTQ short form is a 28-item questionnaire developed to measure five types of childhood maltreatment, i.e., physical, sexual and emotional abuse, as well as physical and emotional neglect. Each scale of the CTQ consist of five items (excluding three minimization items) scored on five-point Likert scales. A total CTQ score is calculated by adding the five scales, leading to a maximum score of 125. Full data on the CTQ was available for 2011 persons.

DSM-IV diagnoses (American Psychiatric Association, 2001) of depressive disorders (DEP; major depressive disorder or dysthymia) and anxiety disorders (ANX; generalized anxiety, social phobia, panic with or without agoraphobia, or agoraphobia without history of panic) were determined by means of the Composite Interview Diagnostic Instrument (CIDI), which was administered by trained research staff. The CIDI has high reliability and validity [32]. CIDI data from four waves, including 6 years of follow-up and retrospective diagnostic information were used to examine lifetime occurrence of depression and/ or anxiety disorder.

To examine more underlying vulnerability to depression and anxiety, the revised Leiden Index of Depression Sensitivity (LEIDS-R [33], *n* = 2267) and the Anxiety Sensitivity Index (ASI [34], *n* = 2233), acquired during the first NESDA wave, were used. The LEIDS-R is a measure of cognitive reactivity to sad mood. The questionnaire consists of 34 items with a five-point Likert scale, ranging from ‘not at all’ (0) to ‘very strongly’ (4). Examples of items are “When I feel down, I lose my temper more easily” or “In a sad mood I do more things that I will later regret”. The ASI measures the degree to which subjects fear anxiety symptoms. The questionnaire consists of 16 items with a five-point Likert scale, ranging from ‘hardly’ (1) to ‘very much’ (5). Examples of items are “It scares me when I am unable to keep my mind on a ‘task’” or “It scares me when my heart beats fast”. For the current study the total scales of the LEIDS-R and ASI were examined.

### Genotyping and imputation

Data on the four OXTR SNPs of interest were extracted from a dataset of a larger GWAS project including samples from NESDA. Methods for biological sample collection and DNA extraction [35] and imputation method and quality control (QC) checks [36] have been extensively described. Briefly, genotyping has been done on multiple chip platforms (Affymetrix-Perlegen 5.0 and Affymetrix 6.0) for several partly overlapping subsets, and QC has been performed within, as well as between platforms and subsets. Subsequently, data were imputed using the 1000 Genomes phase 1 INTEGRATED RELEASE version 3 ALL panel. Post-imputation QC included Minor Allele Frequencies (MAF) >0.01, INFO >0.4 and Hardy Weinberg Equilibrium (HWE) *p* > 0.00001. One of the selected OXTR SNPSs (rs1042778) did not pass post-imputation QC, and was not included in further analyses. The *R* ^2^ values for rs2254298, rs53576 and rs2268498 were, respectively, 0.94, 0.76 and 0.77.

### Statistical analyses

ANOVAs and Chi square tests were used to determine between-group differences in demographic and clinical features. To estimate direct and interaction effects of the OXTR SNPs and CM on lifetime depression and/or anxiety occurrence, logistic regression analyses were performed with lifetime diagnostic status as dependent variable (0 = control group, 1 = DEP and/ or ANX). Gender (0 = male, 1 = female), age and years of education were included in each model as covariates, followed by the OXTR SNPs in three separate analyses to examine their individual direct effects on diagnostic outcome. Due to the low frequency of AA homozygotes of SNPs rs53576 and rs2254298 (see Table 1) the SNP distributions were dichotomized for analyses by comparing A-allele carriers (AA and AG genotypes, coded as 1) to the GG genotypes (coded 0). For rs2268498, the C-allele carriers (CC and CT genotypes, coded 1) were compared to the TT genotype (coded 0). This way we tested dominant models for previously found risk alleles in all three SNPs. In a next analysis CM assessed in the interview (0 = no CM, 1 = any type of CM) was entered to examine its direct effect. Lastly, the interactions between each SNP and CM were added to the logistic regression models, including the main effects of the concerning SNP and CM. To control for population stratification, three ancestry-informative PCs derived from Dutch GWA data [37] were included in the analyses as covariates, but controlling for the PCs did not affect any of the results. Associations between predictors and diagnostic status are expressed as odds ratios (ORs) with 95% confidence intervals (CI).

**Table 1:** Demographic characteristics, maltreatment history and OXTR genotypes for healthy controls and per diagnostic group

Characteristic	Control (*n* = 387)	DEP- (*n* = 428)	ANX- (*n* = 243)	DEP + ANX (*n* = 1509)
% Female	58.7	65.4	62.6	69.1
Age in years (SD)	43.0 (14.3)	42.6 (12.9)	41.9 (14.1)	41.9 (12.5)
Education in years (SD)	13.1 (3.3)	12.5 (3.2)	12.4 (3.2)	11.8 (3.3)
% CM on interview	17.6	38.7	34.3	57.6
CTQ total score (SD)	33.4 (9.7)	39.4 (13.5)	37.2 (11.9)	43.9 (14.9)
LEIDS-R (SD)	15.7 (12.3)	30.1 (17.0)	24.7 (15.2)	42.4 (17.6)
ASI (SD)	23.0 (5.2)	25.5 (7.1)	30.1 (9.5)	32.6 (9.6)
OXTR SNP rs53576				
% AA	12.1	10.0	12.8	12.1
% AG	46.3	47.9	49.4	45.9
% GG	41.6	42.1	37.9	41.9
OXTR SNP rs2254298				
% AA	1.0	0.5	0.4	0.4
% AG	8.0	16.1	9.1	11.1
% GG	91.0	83.4	90.5	88.5
OXTR SNP rs2268498				
% CC	20.9	18.0	21.8	20.7
% CT	54.0	53.5	49.8	49.1
% TT	25.1	28.5	28.4	30.2

*CM* childhood maltreatment, *CTQ* Childhood Trauma Questionnaire, *DEP* depressive disorder, *ANX* anxiety disorder, - no comorbid depressive or anxiety disorder, *OXTR* oxytocin receptor, *SNP* single nucleotide polymorphism, *LEIDS-R* revised Leiden Index of Depression Sensitivity, *ASI* Anxiety Sensitivity Index

To examine whether the OXTR SNPs would be differentially associated with either DEP or ANX, we repeated these logistic regression analyses by separately comparing the groups with DEP (including comorbid DEP + ANX; *n* = 1937) and ANX (including comorbid DEP + ANX; *n* = 1752) to the control group (*n* = 387). Furthermore, two multiple regression analyses were performed with the LEIDS-R and ASI total scores as outcome measures. In these multiple regression analyses, the three SNPs were separately included as predictors, followed by CM and the interaction between the concerning SNP and CM. Again age, gender, education and the three PCs were analyzed as covariates.

In addition, all above analyses were repeated with the CTQ total score a measure of CM. The CTQ data were log-transformed to reduce non-normality, multiplied by ten for interpretation purposes, and centralized to perform the interaction analyses. Lastly, possible specific contributions of the different types of maltreatment were explored by repeating all analyses with the four CM subtypes from the interview and the five CM subtypes from the CTQ.

The logistic and multiple regression analyses were performed in SPSS version 23. In addition, Hardy–Weinberg equilibrium, MAFs, pairwise LD between SNPs, and haplotype association analysis using case–control samples were computed using Haploview 4.2 software. All together 30 interaction effects (three SNPs times five outcomes times two measures of CM) were examined and correction for multiple comparison is therefore warranted. However, the five diagnostic outcomes are highly correlated, as well the two CM measures, and Bonferroni correction was therefore only applied to the three SNPs in the main analyses. Hence, the *p* value for statistical significance was set at 0.017 for the main analyses, and for the explorative analyses with the five different CM sub-types at 0.0034.

## Results

### Sample characteristics

In total 2567 participants (86.1%) were selected from the NESDA sample, with a mean age of 42.2 years [standard deviation (SD) ± 13.0] and 33.7% (*n* = 866) males. Table 1 shows demographic characteristics, CM history (interview and CTQ based) and genotype status per diagnostic group. People with a DEP and/ or ANX disorder reported a lower number of years of education (*p*s < 0.02), and higher levels of abuse (*p*s < 0.001 for the interview, *p*s < 0.05 for the CTQ) than the control sample. Within the comorbid DEP + ANX group even more than 50% reported any type of abuse. The DEP only (DEP-) and DEP + ANX samples included more women than the control group (*p*s < 0.05). People who reported any type of CM were more often female, older, and attained less years of education than people who did not report CM (*p*s ≤ 0.001), hence gender, age and education were included in all analyses as covariates.

All diagnostic groups showed higher LEIDS-R scores than controls, and all groups significantly differed from each other, with the comorbid group showing the highest scores, followed by DEP, ANX and controls (all *p*’s < 0.001). All diagnostic groups also showed higher ASI total scores than controls, and all groups significantly differed from each other, with the comorbid group showing the highest scores, followed by ANX, DEP, and controls (all *p*’s < 0.001), see Table 1.

In the current full sample, MAF ≥ 0.05, HWE *p* > .10 and Linkage Disequilibrium (LD) between rs2254298 and rs53576 was *d*′ = 0.51, between rs53576 and rs2268498 was *d*′ = 0.78 and between rs2254298 and rs2268498 was *d*′ = 0.30. Variation on the SNPs was independent from CM history (interview and CTQ total scores, *p*s > 0.13), age, gender and education (*p*s > 0.08). With regard to the CM subscales (four for the interview and five for the CTQ), emotional neglect measured with the interview was associated with the GG genotype of rs53576 [*χ* ^2^(1) = 4.18, *p* = 0.04] and with the TT genotype of rs2268498 [*χ* ^2^(1) = 4.43, *p* = 0.04], while emotional neglect measured with the CTQ was associated with the A-allele of rs2254298 [*F*(1,2010) = 4.27, *p* = 0.04]. These findings could be due to chance as 27 comparisons were made (nine scales times three SNPs). However, emotional abuse measured with the interview was also significantly associated with the TT genotype of rs2268498, with a much lower *p* value [*χ* ^2^(1) = 10.3, *p* = 0.001], which suggests the possibility of a gene by environment correlation.

### Associations between SNPs, CM and diagnostic status

No significant main effects of the OXTR SNPs, and no significant interaction effects between the SNPs and CM were found in the prediction of DEP and/or ANX, neither with the interview based score nor with the CTQ (see Table 2). Results were similar when we repeated these analyses for the people with lifetime DEP or ANX versus controls separately (see Table 2).

**Table 2:** The effects of CM (interview and CTQ), the three OXTR SNPs, and their interactions on the presence of any diagnosis (ANX and/or DEP), ANX or DEP versus healthy controls

Outcome comparison	Direct effect		SNP × CM interview		SNP × CM-CTQ	
	OR (95% CI)	*p* value	OR (95% CI)	*p* value	OR (95% CI)	*p* value
DEP and/or ANX vs control						
CM-interv	4.97 (3.76–6.57)	<0.001				
CM-CTQ	2.09 (1.84–2.39)	<0.001				
rs53576_A	1.01 (0.81–1.26)	0.92	1.00 (0.57–1.76)	0.99	1.16 (0.89–1.50)	0.28
rs2254298_A	1.44 (0.99–2.09)	0.06	0.92 (0.37–2.28)	0.85	0.73 (0.49–1.07)	0.11
rs2268498_C	0.81 (0.63–1.05)	0.10	0.98 (0.52–1.83)	0.95	0.93 (0.69–1.24)	0.60
DEP vs control						
CM interv	5.41 (4.08–7.16)	<0.001				
CM-CTQ	2.20 (1.92–2.51)	<0.001				
rs53576_A	0.99 (0.79–1.23)	0.90	1.06 (0.60–1.86)	0.84	1.18 (0.91–1.54)	0.22
rs2254298_A	1.48 (1.01–2.16)	0.04	0.86 (0.34–2.16)	0.75	0.71 (0.48–1.06)	0.09
rs2268498_C	0.80 (0.62–1.02)	0.08	1.01 (0.54–1.91)	0.97	0.97 (0.72–1.30)	0.81
ANX vs control						
CM interv	5.67 (4.27–7.52)	<0.001				
CM-CTQ	2.18 (1.91–2.50)	<0.001				
rs53576_A	1.01 (0.81–1.28)	0.86	0.98 (0.55–1.72)	0.93	1.14 (0.87–1.48)	0.35
rs2254298_A	1.23 (0.87–1.88)	0.21	0.96 (0.38 − 0.243)	0.93	0.74 (0.49–1.10)	0.14
rs2268498_C	0.80 (0.62–1.03)	0.08	1.00 (0.53–1.90)	0.99	0.93 (0.69–1.26)	0.63

The direct effects of the SNPs, CM and their interaction effects (SNP × CM) on the three outcome comparisons were estimated in separate logistic regression models including age, gender, and education as covariates. The models estimating SNP*CM also included the main effects of the concerning SNP and CM

*CM* childhood maltreatment, *CTQ* Childhood Trauma Questionnaire, *DEP* depressive disorder, *ANX* anxiety disorder, *SNP* single nucleotide polymorphism

As previously shown, CM was significantly associated with higher risk for DEP and/or ANX [interview and CTQ, respectively, OR = 4.97; 95% CI (3.76, 6.57), *p* < 0.001; OR = 2.09; 95% CI (1.84, 2.39), *p* < 0.001], which was similar to findings when lifetime DEP or ANX versus controls were analyzed separately (both *p*s < 0.001).

For all three outcome comparisons the CM subtypes from the interview and CTQ were analyzed in interaction with the three SNPs as well. None of the interactions with the CM subtypes reached significance (all *p*s > 0.04, data not shown).

We furthermore examined LD and haplotype associations in each of the three samples (any diagnosis vs. controls, all DEP vs. controls, and all ANX vs. controls). LD was *d*′=0.78 between rs53576 and rs2268498 in all three analyses, but haplotype associations were non-significant.

### Associations between SNPs, CM and depression and anxiety sensitivity

No significant main effects of the OXTR SNPs, and no significant interaction effects between the SNPs and CM were found in the prediction of depression sensitivity or in the prediction of anxiety sensitivity (see Table 3).

**Table 3:** The effects of CM (interview and CTQ), the three OXTR SNPs, and their interactions on depression sensitivity (LEIDS-R) and anxiety sensitivity (ASI)

Outcome	Direct effect		SNP × CM-interview		SNP × CM-CTQ	
	Beta	*p* value	Beta	*p* value	Beta	*p* value
LEIDS-R						
CM interview	0.32	<0.001				
CM-CTQ	0.36	<0.001				
rs53576_A	0.39	0.64	0.01	0.71	−0.004	0.91
rs2254298_A	0.02	0.41	0.02	0.54	0.02	0.30
rs2268498_C	−0.01	0.50	−0.004	0.92	0.05	0.26
ASI						
CM interview	0.13	<0.001				
CM-CTQ	0.16	<0.001				
rs53576_A	0.03	0.11	0.01	0.72	−0.02	0.56
rs2254298_A	0.01	0.54	0.05	0.12	0.009	0.71
rs2268498_C	−0.009	0.68	0.01	0.77	0.009	0.83

The direct effects of the SNPs, CM and their interaction effects (SNP*CM) on the two outcomes were estimated in separate multiple regression models including age, gender, and education as covariates. The models estimating SNP*CM also included the main effects of the concerning SNP and CM

*CM* Childhood Maltreatment, *CTQ* Childhood Trauma Questionnaire, *SNP* single nucleotide polymorphism

As expected, a history of CM was associated with higher scores on the LEIDS-R (interview and CTQ, respectively, *β* = 0.32, *p* < 0.001, *β* = 0.36, *p* < 0.001) and with higher scores on the ASI (interview and CTQ, respectively, *β* = 0.13, *p* < 0.001, *β* = 0.16, *p* < 0.001).

For both the LEIDS-R and ASI the CM subtypes from the interview and CTQ were analyzed in interaction with the three SNPs as well. None of the interactions with the CM subtypes reached significance (all *p*s > 0.01, data not shown).

## Discussion

In the current study we replicated the effect that CM by itself is a strong predictor of both depression and anxiety diagnoses and sensitivity, as has been shown before (e.g. [2]). However, no indications were found for moderation of these associations by three literature-based OXTR SNPs (rs2254298, rs53576 and rs2268498). Previous studies reporting interactions between OXTR SNPs and stressful early life experiences examined symptom levels of anxiety and depression in community samples, or (endo)phenotypic characteristics of anxiety and depression [22–25], while in the current study we examined a large clinical sample with lifetime depression and/ or anxiety disorders. Non-clinical or underlying characteristics of depression and anxiety may be more suitable to examine in genetic risk studies, as they can be defined more precise than the quite broad diagnostic categories of clinical depression and anxiety. However, in the current sample we did not find evidence for gene by environment interactions on more trait related depression and anxiety sensitivity measures either.

Possibly, despite our large clinical sample, this sample size was still too small to detect gene by environment interactions. Recently, discussions have been ongoing about the validity of many candidate-gene by environment interaction studies in psychiatry [4, 38, 39], and the importance of large replication studies with clear disease and environment measures in this area is stressed [4]. However, a strength of our study was that we used both dichotomous and continuous outcomes to assess depression and anxiety diagnoses and sensitivity, testing therefore the gene by environment interaction both with a multiplicative (prone to statistical artifacts [39]) and additive scale. Also, to increase the reliability of the assessment of childhood maltreatment both interview and self-report measures were included.

Besides the negative findings regarding the gene–environment interactions, we did not find any main effects of the three OXTR SNPs on lifetime clinical depression or anxiety either. The null findings regarding associations between OXTR SNPs and depression are in line with recent GWA studies [10, 11]. Only a small candidate gene study by the Costa and colleagues [12] has so far shown associations between OXTR SNPs and clinical diagnoses of depression. The null findings regarding associations between OXTR SNPs and anxiety are also in line with a recent GWA study [14]. In contrast, quite a few candidate gene studies, often in non-clinical samples, have shown main effects of OXTR genes on underlying characteristics of depression and anxiety (e.g. [15–21]), which we were not able to replicate. While one of the strengths of the current study is the clinical assessment of lifetime depression and anxiety diagnoses in addition to underlying characteristics of depression and anxiety, within clinical samples the environment may play a very large role in the etiology of the disorders. For example, more than half of the people in the current comorbid sample (which constituted the largest part of the current clinical sample) reported a history of childhood maltreatment. This indicates the need to include very large and homogeneous diagnostic groups to disentangle possible genetic contributions to phenotypic outcomes [40, 41]. Also, due to the inherently small effects of SNPs on such major diagnostic outcomes, a promising route in this area might be studies in which polygenic risk scores are examined in interaction with environmental influences [40].

One unexpected significant finding was the association between the reported experience of emotional abuse and the TT genotype of SNP rs2268498. This indicates a gene–environment correlation, which could have multiple causes, e.g., the genotype could be associated with behavior that may evoke abusive behavior by the parents, or the genotype could be associated with abusive characteristics and genetic inheritance then leads to associations with abuse experiences in the offspring. Some studies have shown this SNP to be associated with social and empathic behaviors [42–44], and could be of interest to study further in the context of maltreatment. Intriguingly, the presence of gene–environment correlation may have increased the likelihood of detecting spurious gene–environment interactions, none of which were significant in the current study. This reinforces the finding of the absence of interplay between CM and the OXTR gene variants in depression and anxiety.

We did find strong associations between childhood maltreatment and both diagnostic measures of depression and anxiety as well as depression and anxiety sensitivity measures. This is a consistent finding in the literature [1–3], and indicates the importance of studying factors that are involved in both risk and resilience with regard to the psychosocial effects of childhood maltreatment. Knowledge on specific genetic markers of vulnerability or susceptibility can point to biological mechanisms that could be of interest for personalized diagnostic and therapeutic approaches.

### Limitations and future directions

While the current sample size is quite large for a candidate gene study, the study may still be underpowered for investigating gene by environment interactions [4, 38]. Thus, the results remain to be replicated, both in Caucasian samples and other ethnical groups, as ethnicity is also known to influence the role of the OXTR [45]. And even though we measured childhood maltreatment quite extensively, the measurement of stressful early life experiences could be broadened to other childhood traumas and later life trauma, or by using multiple informants in addition to only self-report, so that social desirable answering, recall bias and memory problems can be reduced. Furthermore, there are other OXTR SNPs that were not part of the current investigation (see e.g. [16, 18, 22]) that may be involved in the development of depression or anxiety. The current SNPs rs2254298 and rs53576 are located on one of the introns of the OXTR gene on chromosome 3, while rs2268498 is located in the promotor region of the gene. These SNPs are part of haplotype blocks that have previously been examined in association with depression and anxiety outcomes [18, 22], and are most often mentioned in the literature concerning depression, anxiety and related psychological outcomes [8]. We therefore considered these SNPs to be most important to examine in the current context, but future studies may opt for gene-set analyses or pathway analyses involving the oxytocin system.

It may furthermore be the case that the OXTR gene does not (only) moderate the effect of early life stress on later psychological outcomes, but that certain OXTR gene variants are only expressed under stressful circumstances. Such gene–environment interactions could be explained by epigenetic effects, which encompass processes by which environmental factors influence expression of genes via epigenetic modifications. Epigenetic modulations of the OXTR gene have indeed been shown in rodents and humans [46–51]. For example, Unternaehrer et al. [49] showed that low maternal care in human childhood was associated with greater OXTR gene methylation. Depression and anxiety in turn have been associated with higher levels of OXTR methylation, and these were moderated by the OXTR SNP rs53576 [47, 48, 51]. More methylation of the OXTR gene is assumed to result in reduced OXTR expression [46]. Hence, epigenetic processes, under the influence of the environment, may play a crucial role in the expression of the OXTR gene, and through these mechanisms affect behavioral outcomes. Hence, future studies could include measurements of epigenetic modulations of the OXTR gene, mRNA and OXTR expression, or sensitivity to oxytocin, as well as more elaborate (endo)phenotypes and underlying characteristics of depression and anxiety. Interactions with other hormonal systems, including their genetic predispositions, should be elucidated as well, as the oxytocin system may interact with monoaminergic systems, inflammatory processes, and growth factors in developing stress-related disorders [52].

Lastly, while in recent years clinical trials have been performed to examine the effects of oxytocin administration on anxiety and depressive disorders [53, 54], the role of individual differences in genetic makeup of the oxytocin system and the role of early life trauma in both the etiology of these disorders, and in the possible moderation of treatment effects are still unclear. Hence, the possible clinical application of genetically informed treatments with regard to the oxytocin system still remain to be elucidated.

## Conclusions

In sum, while the OXTR gene has been found to play a role in social processes and underlying characteristics of both anxiety and depression, there is little evidence for a role of the OXTR in the etiology of clinical expressions of anxiety and depression. That is, GWA studies have not implicated a role for the OXTR, and only one candidate gene study so far has shown associations of depression with OXTR SNPs. While studies in non-clinical population suggest the OXTR gene may interact with early life stress in the development of depressive and anxiety symptomatology, here we show that OXTR SNPs are not involved in moderating the effects of early life stress on lifetime anxiety and depressive disorders. These findings stress the importance of replication research within the domain of oxytocin research, both in the general population as well as in clearly established clinical samples.
